# Nephron-sparing surgery for primary mucinous adenocarcinoma of renal pelvis in horseshoe kidney: A case report

**DOI:** 10.1097/MD.0000000000033410

**Published:** 2023-03-24

**Authors:** Dapeng Li, Wei Zhang, Jun Wang, An Wang, Mingming Wu, Yangguang Wei

**Affiliations:** a Department of Urology, Rongcheng People’s Hospital, The Affiliated Hospital of Jining Medical University, Weihai, Shandong, China.

**Keywords:** adenocarcinoma, horseshoe kidney, mucinous, renal pelvis

## Abstract

Mucinous adenocarcinoma of the renal pelvis is extremely rare in malignancy of the renal pelvis, and <100 cases have been reported so far. However, horseshoe kidneys appear to be more prone to this mucinous gland metaplasia. Due to the lack of a specific diagnostic basis, it is difficult to confirm the diagnosis before surgery, which is easy to cause misdiagnosis.

**Patient concerns:**

We report a 64-year-old male patient who found a cystic mass in the left kidney during physical examination. CT examination showed a horseshoe kidney with a cystic mass 9.5 × 8.0 cm in front of the left kidney, lacking obvious diagnostic features.

**Diagnosis:**

It was misdiagnosed as cystic teratoma before the operation, and was diagnosed as mucinous adenocarcinoma of the renal pelvis through pathological examination after the operation.

**Interventions:**

Resection of the tumor by nephron-sparing surgery and postoperative chemotherapy.

**Outcomes:**

No tumor recurrence was found at 6 years of follow-up. After 7 years, the patient had multiple metastases in the abdominal wall and peritoneum, and no tumor recurrence was found in the urinary system. The patient received chemotherapy again and survived well.

**Lessons:**

The prognosis of nephron-sparing tumor resection for MRAP is not significantly different from that of radical nephroureterectomy + bladder cuff excision. Because it can reduce the risk that patients can not tolerate follow-up chemotherapy due to abnormal renal function after surgery, which may be more beneficial in patients with kidney abnormalities or chronic disease.

## 1. Introduction

Malignant tumors of the renal pelvis usually originate from urothelial cells, of which urothelial carcinoma accounts for >90%, followed by squamous cell carcinoma and adenocarcinoma. Adenocarcinoma is rare and accounts for <1% of tumors in the renal pelvis. It is generally believed that glandular metaplasia of renal pelvis mucosa cells occurs under the stimulation of chronic inflammation, such as renal calculus, hydronephrosis, infection, etc. Mucous cystadenoma is a benign disease, and approximately 30% of it will become mucinous adenocarcinoma.^[1]^ In 2017, Xiang H et al^[2]^ summarized the data of a total of 22 cases of renal mucinous cystadenoma reported and found that 3 of them were horseshoe kidney patients. Primary mucinous adenocarcinoma of the renal pelvis (MARP) is an extremely rare malignant tumor with fewer than 100 cases reported to date.^[3]^ The horseshoe kidney is accompanied by MARP, which is the third case reported since the second case reported by Higgins A et al in 2017.^[4]^ The treatment of MARP mostly adopts radical nephroureterectomy (RNU) + bladder cuff excision (BCE) with postoperative adjuvant chemotherapy, and the prognosis is usually poor. In this case, the patient underwent tumor resection with nephron-sparing and postoperative adjuvant chemotherapy and achieved good results.

## 2. Case report

**Figure 1.**
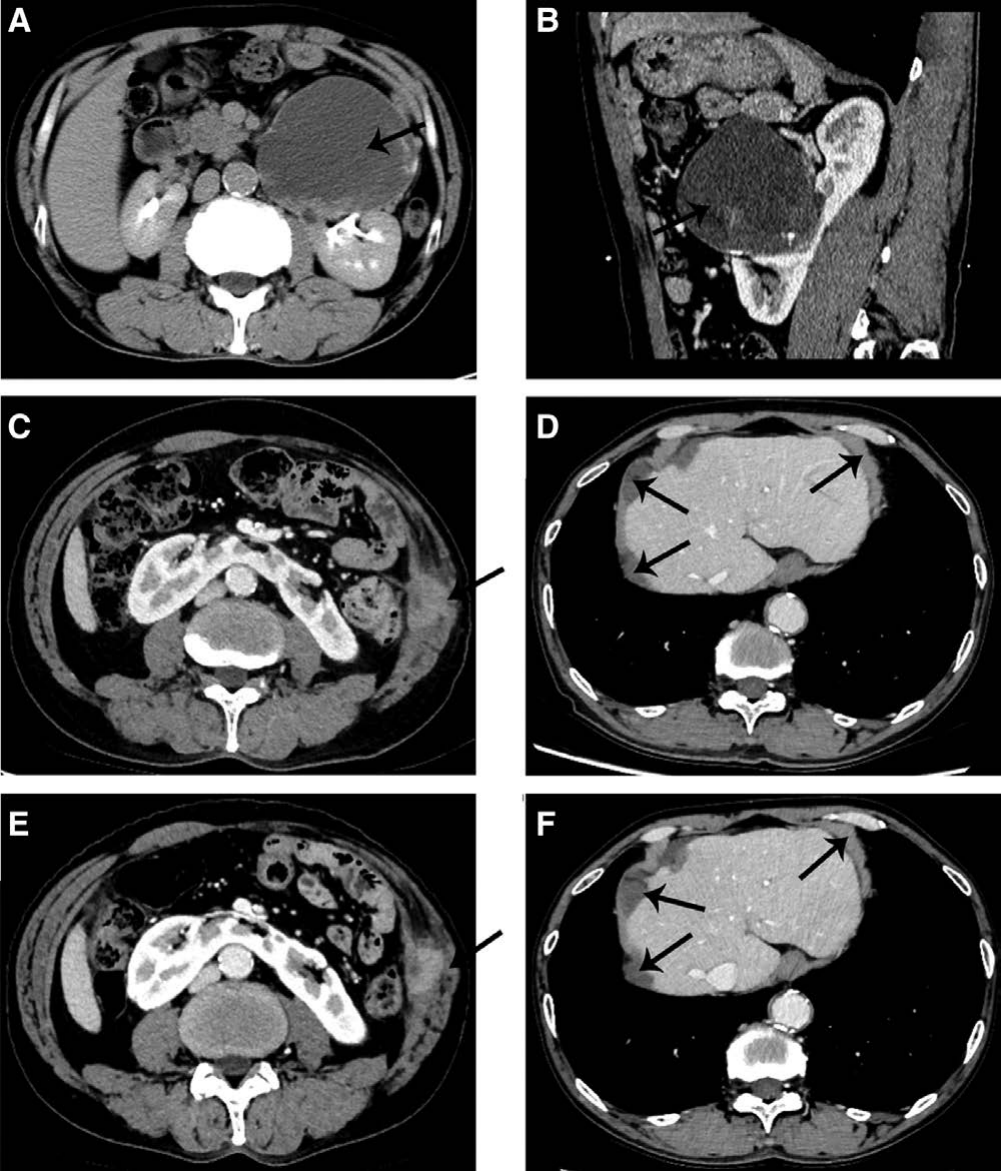
(A) A huge cystic mass in front of the left kidney of the horseshoe kidney, calcification can be seen in it, and the urocontrast agent is not significantly filled. (B) The sagittal position shows that the mass is closely related to the kidney. (C) Weak enhancement mass can be seen in the left abdominal wall muscle layer. (D) Multiple low-density nodules can be seen in the perihepatic peritoneum. (E) The tumor in the muscular layer of the left abdominal wall is slightly smaller than before. (F) Perhepatic peritoneal low-density nodules did not change significantly.

**Figure 2.**
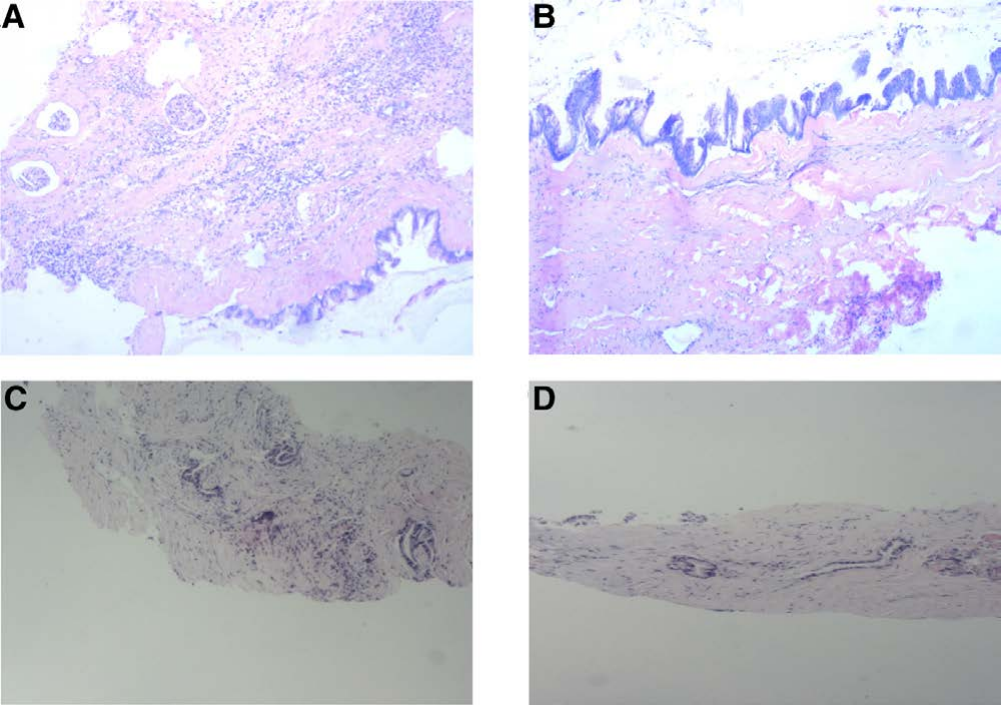
(A) The coating epithelium is differentiated into mucus, and glomeruli are visible underneath. (×200). (B) Mucous differentiation of surface epithelium, cell atypia. (×200). (C) Dysplastic glands seen in connective tissue. (×100). (D) Mucus differentiation can be seen in glands. (×100).

The patient, a 64-year-old male, was hospitalized in the Department of Urology of the Rongcheng People’s Hospital in March 2015 due to a physical examination of the left anterior renal cystic occupancy. CT examination (shown in Fig. 1A and B) found that the patient had a horseshoe kidney, and the left renal cystic mass (9.5 × 8.0 cm) was diagnosed as cystic teratoma. The tumor was found to be located in the space between the retroperitoneum and the front of the left kidney during the open surgery. The pseudo capsule was seen outside the tumor, and part of the tumor adhered to the renal parenchyma. Part of the renal tissue was removed and the tumor was completely removed. The wound of the kidney was carefully hemostasized and sutured. Postoperative incision of the specimen showed that the tissue in the tumor was jelly-shaped and calcified tissue was visible. The postoperative pathology showed mucinous adenocarcinoma, which tended to be of renal pelvic origin and involved renal tissue (shown in Fig. 2A and B). The patient further received chemotherapy with “gemcitabine + cisplatin” for 4 cycles. No tumor recurrence was found after 6 years of follow-up. In July 2022, the patient came to the clinic due to the discovery of a mass on the left abdominal wall, and a CT examination (shown in Fig. 1C and D) showed that the mass of the left abdominal wall, the right peritoneal nodule, and multiple nodular protrusions at the liver margin. The biopsy of abdominal wall masses shows invasion of the adenocarcinoma (shown in Fig. 2C and D). Immunohistochemistry: PAX-8(−), PSA(−), TTF-1(−), CK7(+), CK20(−), Villin(+), CDX-2(+), and Her-2(+). Further PET-CT scanning showed that there was no abnormal density in the operation area of the left renal malignant tumor. The perihepatic, right omentum, pelvic peritoneum, left abdominal wall multiple low-density, soft tissue density nodules and masses, FDG metabolism increased to varying degrees, consider perihepatic peritoneum, omentum, pelvic peritoneum, left abdominal wall multiple metastases. Due to the inability to tolerate the side effects of cisplatin, 6 cycles of chemotherapy with docetaxel and carboplatin were given. Now the patient is generally in good condition. In February 2023, a CT reexamination showed a soft tissue mass on the left abdominal wall and a density shadow of the right subperitoneal soft tissue, with a slightly smaller range than before (shown in Fig. 1E and F).

## 3. Discussion

Non-urothelial tumors of the renal pelvis usually occur due to mutations after metaplasia of urothelial cells.^[2]^ Some scholars believe that MARP may originate in the isolated part of the renal pelvic epithelium in the renal parenchyma, especially in abnormal kidneys such as horseshoe kidneys.^[5]^

Since the incidence rate of MARP is extremely low. At present, there is no unified guidance for its treatment, but most scholars believe that radical resection of tumors can benefit patients. Referring to the treatment standard of urothelial carcinoma of the renal pelvis, they believe that the standard operation should be RNU + BCE.

There is no characteristic difference between the imaging of MARP and complex renal cyst, renal calculus with hydronephrosis, and other diseases. Although some scholars found that the serum CEA and CA19-9 levels in patients with this disease increased, which may provide a reference for the diagnosis of this disease.^[6]^ But the diagnosis of most patients mainly depends on the pathological diagnosis after the operation. For patients with MARP, it is difficult to make a clear diagnosis before the operation. This makes most of the patients due to preoperative misdiagnosis and the best surgical plan is not used during surgery. From the data of 30 patients summarized by Li H et al,^[7]^ only 4 patients were treated with near-RNU + BCE, and the other patients were only treated by nephrectomy or tumor resection. From the known follow-up results, the average survival of the 2 patients treated with RNU + BCE was (15 ± 12.73) M, and the 18 patients who underwent nephrectomy or tumor resection, except for 1 case who died perioperatively 3 days after surgery, the average survival time was (14.47 ± 14.13) M, and there was no statistically significant difference between the 2 groups (*t* = 0.05, *P* = .96).

We consider the reason why there is no obvious difference between the two treatment results. It may be that the obstruction of the renal pelvis junction caused the formation of hydronephrosis or cystic lesions in the patient earlier, which is conducive to preventing tumor cells from excreting with urine and causing the tumor implantation in the ureter and bladder. In fact, according to the retrieved literature, MARP is rarely found in the ureter or bladder. Therefore, complete resection of the tumor or kidney can also achieve the same effect. In addition, some follow-up adjuvant treatment may be required after the operation. In May 2022, Liu DHW et al^[8]^ proved through research that neoadjuvant chemotherapy can improve the survival rate of patients with esophageal mucinous adenocarcinoma. That had also improved people’s confidence in the effectiveness of chemotherapy for MARP. In addition, the research of Ye S et al^[9]^ shows that intraperitoneal hyperthermic perfusion chemotherapy has a good effect on peritoneal pseudomyxoma. This also increases the treatment methods and treatment confidence for patients with intraperitoneal metastasis of MARP.

## 4. Conclusion

In this case report, the patient underwent nephron-sparing surgery to preserve the patient’s renal function as much as possible and received chemotherapy after the operation. At present, 8 years after surgery, the patient has tumor metastasis and recurrence, which may be caused by tumor rupture during the operation and tumor dissemination and implantation, he survives well after chemotherapy again. The prognosis of nephron-sparing tumor resection for MRAP is not significantly different from that of RNU + BCE. Because it can reduce the risk that patients can not tolerate follow-up chemotherapy due to abnormal renal function after surgery, which may be more beneficial in patients with kidney abnormalities or chronic disease.

## Author contributions

**Conceptualization:** Dapeng Li.

**Data curation:** Dapeng Li, Wei Zhang, An Wang, Mingming Wu, Yangguang Wei.

**Formal analysis:** Dapeng Li, Wei Zhang, An Wang.

**Funding acquisition:** Dapeng Li, Wei Zhang.

**Investigation:** Dapeng Li, Mingming Wu.

**Methodology:** Dapeng Li, Jun Wang.

**Writing – original draft:** Dapeng Li, Wei Zhang, Jun Wang, Mingming Wu, Yangguang Wei.

**Writing – review & editing:** Dapeng Li, Jun Wang, An Wang.
